# Measurement of oxygen consumption rate in mouse aortic tissue

**DOI:** 10.1093/biomethods/bpaf031

**Published:** 2025-04-24

**Authors:** Zhen Zhou, Ripon Sarkar, Jose Emiliano Esparza Pinelo, Alexis Richard, Jay Dunn, Zhao Ren, Callie S Kwartler, Dianna M Milewicz

**Affiliations:** Division of Medical Genetics, Department of Internal Medicine, The University of Texas Health Science Center at Houston McGovern Medical School, Houston, TX, 77030, United States; Division of Medical Genetics, Department of Internal Medicine, The University of Texas Health Science Center at Houston McGovern Medical School, Houston, TX, 77030, United States; Department of Anesthesiology, The University of Texas Health Science Center at Houston McGovern Medical School, Houston, TX, 77030, United States; Division of Medical Genetics, Department of Internal Medicine, The University of Texas Health Science Center at Houston McGovern Medical School, Houston, TX, 77030, United States; Division of Medical Genetics, Department of Internal Medicine, The University of Texas Health Science Center at Houston McGovern Medical School, Houston, TX, 77030, United States; Cell Analysis Division, Agilent Technologies, 5301 Stevens Creek Blvd, Santa Clara, CA, 95051, United States; Division of Medical Genetics, Department of Internal Medicine, The University of Texas Health Science Center at Houston McGovern Medical School, Houston, TX, 77030, United States; Division of Medical Genetics, Department of Internal Medicine, The University of Texas Health Science Center at Houston McGovern Medical School, Houston, TX, 77030, United States; Division of Medical Genetics, Department of Internal Medicine, The University of Texas Health Science Center at Houston McGovern Medical School, Houston, TX, 77030, United States

**Keywords:** aortic tissue, mitochondrial respiration, oxygen consumption rate, Seahorse extracellular flux

## Abstract

Thoracic aortic aneurysm and dissection (TAD) is a life-threatening vascular disorder, and smooth muscle cell mitochondrial dysfunction leads to cell death, contributing to TAD. Accurate measurements of metabolic processes are essential for understanding cellular homeostasis in both healthy and diseased states. While assays for evaluating mitochondrial respiration have been well established for cultured cells and isolated mitochondria, no optimized application has been developed for aortic tissue. In this study, we generate an optimized protocol using the Agilent Seahorse XFe24 analyzer to measure mitochondrial respiration in mouse aortic tissue. This method allows for precise measurement of mitochondrial oxygen consumption in mouse aorta, providing a reliable assay for bioenergetic analysis of aortic tissue. The protocol offers a reproducible approach for assessing mitochondrial function in aortic tissues, capturing both baseline OCR and responses to mitochondrial inhibitors, such as oligomycin, FCCP, and rotenone/antimycin A. This method establishes a critical foundation for studying metabolic shifts in aortic tissues and offers valuable insights into the cellular mechanisms of aortic diseases, contributing to a better understanding of TAD progression.

## Introduction

The thoracic aorta plays a pivotal role in the cardiovascular system, serving as the primary conduit for blood from the heart to the rest of the body. The major conditions affecting the thoracic aorta are asymptomatic aneurysms in the root or ascending aorta that progressively enlarge until the aorta becomes unstable, leading to acute ascending aortic dissections. These dissections cause sudden death in up to 50% of afflicted individuals but are preventable if the aneurysm is repaired before the aorta dissects [1–3].

Previous studies identified mitochondrial dysfunction in aortic cells as a potential contributor to thoracic aortic diseases [4–6]. *Fbn1^C1041G^*^/+^ mice form thoracic aortic aneurysms and show decreased expression of mitochondrial complex subunits and genes related to mitochondrial biogenesis, suggesting mitochondrial dysfunction is associated with aortic aneurysm formation [6]. Studies on aortas from patients with *FBN1* pathogenic variants and Marfan syndrome identified similar mitochondrial dysfunction [7].

The Agilent Seahorse XFe bioanalyzer was designed to precisely quantify bioenergetics profiles by measuring the oxygen consumption rate (OCR) and extracellular acidification rate (ECAR) in real-time. OCR reflects mitochondrial respiration and ECAR serves as an indicator of glycolytic activity in live cells. These measurements can be performed in cultured cells, and OCR can also be assessed in isolated mitochondria [8, 9]. However, the *in vitro* method does not reflect a true state of mitochondrial function *in vivo* because isolated cells lose their native cellular environment.

The Agilent Seahorse XFe bioanalyzer also allows the measurement of OCR from tissues [10–17]. However, measuring the OCR in tissue is challenging due to both biological and technical variabilities, such as that caused by the tissue movement during drug injection and mixing. Agilent introduced an XFe islet capture microplate for spheroids, organoids, and other three-dimensional cultures, in which a capture screen is placed to imprison the tissue sample at the bottom of the microplate well. We used the XFe islet capture microplate to measure the OCR in aortic tissue *ex vivo*. The thickness of normal mouse aortic tissue is approximately 50–100 µm, which fits well in between the capture screen and the bottom surface of the well and holds the tissue undisrupted. Thus, a mitochondrial stress test can generate bioenergetic profiles of basal respiration, ATP-linked respiration, non-ATP-linked respiration, maximal respiration, spare respiratory capacity, and non-mitochondrial oxygen consumption. Mitochondrial respiration was previously reported for aortic tissue [11], but there were limitations in terms of drug concentrations, tissue size, and data normalization.

In this study, we generate a rigorous and reproducible protocol for measuring OCR in mouse aortic tissue, and our results show no sex differences of OCR values between ascending or descending aortas.

## Materials and methods

### Animals

All experimental procedures were designed in accordance with National Institutes of Health guidelines and approved by the Animal Welfare Committee and the Center for Laboratory Animal Medicine and Care at the University of Texas Health Science Center at Houston. Seven-to-eight-week-old male and female C57BL/6J wild-type mice were purchased from Jackson Laboratory (Bar Harbor, ME, USA), and in-house breeding was performed to get mice for experiments. All pups were housed on a 12-h light/dark cycle with ad libitum access to food and water. At postnatal (P) day 28 or 32, or 10 weeks of age, both male and female pups were randomized and subjected to each assay.

### Preparation of sensor cartridge

1. Add 1 mL of XF calibrant solution (Agilent, catalog# 100840-000) to each well of the plate and submerge the XFe24 sensor cartridge (Agilent, catalog# 103518-100) into the plate.
2. Incubate the sensor cartridge for overnight (at least 3 h) at 37°C in a non-CO_2_ humidified incubator.

### Preparation of assay medium

Assay medium was freshly prepared with XF DMEM basal medium (Agilent, catalog# 103575-100), 1 M glucose stock (1%, final concentration 10 µM; Agilent, catalog# 103577-100), 100 mM pyruvate stock (1%, final concentration 1 µM; Agilent, catalog# 103578-100), and 200 mM glutamine (1%, final concentration 2 µM; Agilent, catalog# 103579-100).

### Preparation of 2-mm aortic tissue discs

**Figure 1.**
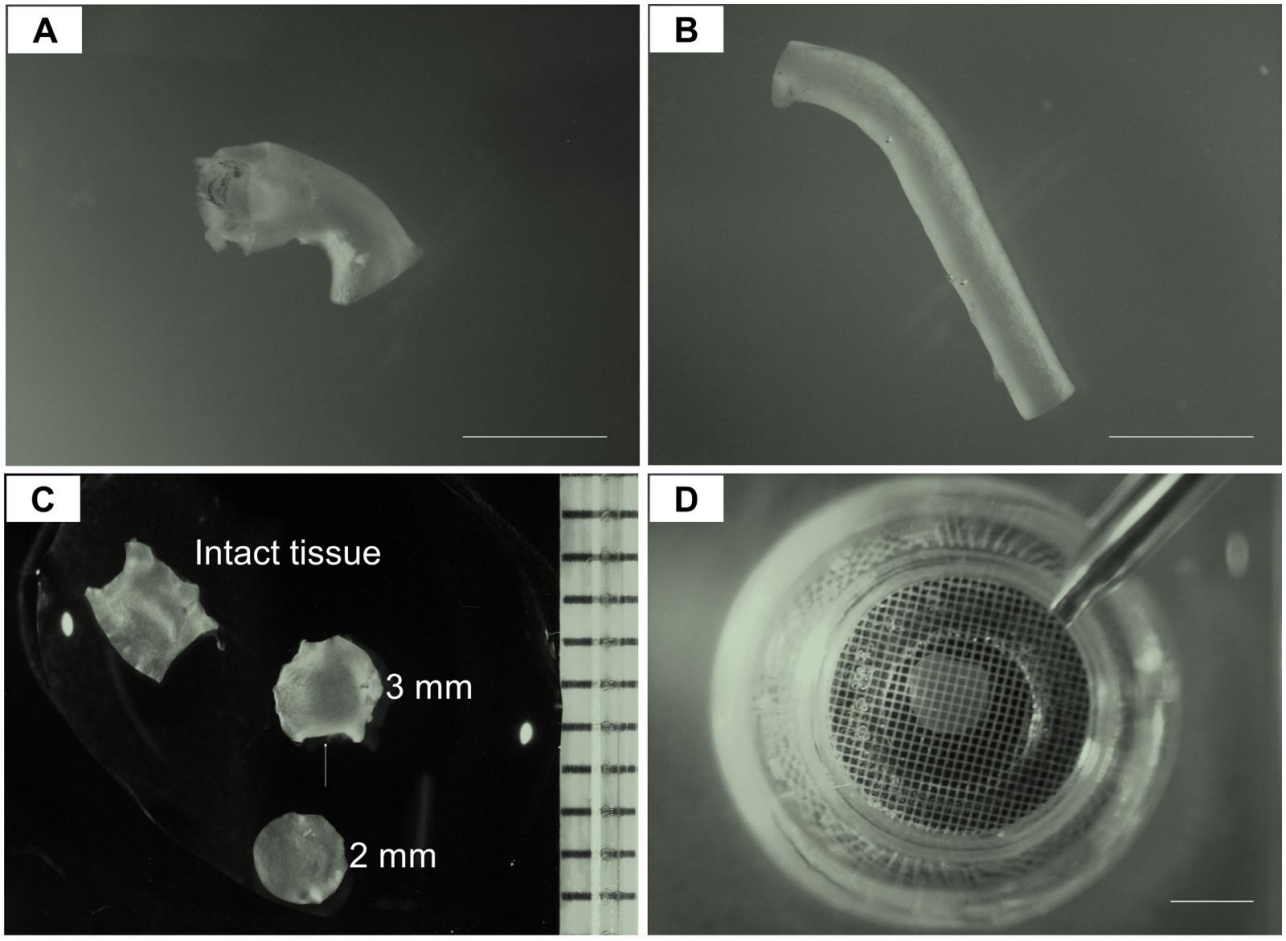
Representative images of the ascending and descending thoracic aortic tissues from mice at the age of 28 days. A. Ascending aorta; B. Descending thoracic aorta; C. Representative images of an intact, longitudinally opened ascending aorta, an irregular 3-mm tissue disc (white arrow indicating the defect), and a regular 2-mm tissue disc; D. 2-mm aortic tissue disc in a Seahorse islet plate. Scale bar = 2 mm

1. After intraperitoneal injection with Avertin (2.5%, 350 mg/kg; Sigma-Aldrich, catalog# T48402-25g), euthanized animals were perfused with 10 mL ice-cold PBS (pH=7.4) through the left ventricle under physiological pressure.
2. Ascending and descending thoracic aortas were excised (**Fig. 1A-1B**), longitudinally opened [18–20], and submerged in ice-cold Hanks' Balanced Salt Solution (HBSS) without Ca^2+^ or Mg^2+^ (Thermo Fischer Scientific, catalog# 14170112).
3. Freshly collected segments of ascending and descending aorta were gently washed with ice-cold assay medium for 3 times, each for 3 minutes.
4. After the last wash, a 2-mm aortic tissue disc (**Fig. 1C**) was collected using a 2-mm disposable biopsy punch (World Precision Instruments, catalog# 501817).

Notes: 1. To maintain cell viability, it is crucial to complete the tissue preparation procedures within 2 h of dissecting the aortic tissue. 2. Avoid using serum in HBSS or assay medium, as it can significantly increase baseline OCR values and compromise the accuracy of the results.

### Place aortic tissue discs into islet capture microplate

1. Add 500 μL assay media to each well of a Seahorse XF24 Islet Capture Microplate (Agilent, catalog# 101122-100) and prewarm the plate at 37°C without CO_2_.
2. Place the aortic tissue disc into the designated well, and secure it in the center of the bottom using the islet screen, with the web side facing up (**Fig. 1D**).
3. Cover the blank wells with the islet screens in the same manner.
4. Inspect each tissue punch to ensure it is placed at the bottom of the well; If not, gently flush the tissue with a 200 µL tip, avoiding the generation of air bubbles.
5. Incubate the plate for 1 h at 37°C without CO_2_.

Note: The orientation of the tissue disc does not affect the OCR value, so either the intima or adventitia can face up.

### Preparation of drugs

1. During the 1-h tissue incubation, prepare the drugs (**Supplementary Table 1**) by dissolving the lyophilized powders in assay medium using the Seahorse XF Cell Mito Stress test kit (Agilent, catalog# 103015-100), including oligomycin, carbonyl cyanide-4 (trifluoromethoxy) phenylhydrazone (FCCP), and rotenone/antimycin A (Rot/AA).
2. Add each drug in the specific volume to the designated port on the sensor cartridge, then return the plate to the incubator.

### Measurement of OCR

1. Select a default Cell Mito Stress Test template in the Wave software, fill in the basic information and the optimized measurement settings (**Supplementary Table 2**). Click “Run Assay” and place the sensor cartridge with drugs on the platform of the XFe24 Analyzer.
2. When the calibration is completed with all 24 sensors working normally, replace the utility plate with the islet microplate containing the aortic tissue discs on the platform of the machine, and start the assay.
3. When the measurement is completed, take out the islet plate and wash the aortic tissue discs with cold PBS for 3 times, each for 3 minutes.
4. Start the DNA extraction and quantification immediately, or store the tissues in −80°C for future assessment.

### DNA extraction and quantification

DNA of each aortic tissue disc was extracted using the DNeasy Blood and Tissue Kits for DNA Isolation (QIAGEN, catalog# 69506).

1. Place each tissue disc in a 1.5-mL microcentrifuge tube, add 90 μL Buffer ATL and 10 μL proteinase K. Mix by vortexing and incubate at 56°C until the tissues are completely lysed (≥ 2 h). Vortex occasionally during incubation, and vortex for 15 seconds before proceeding to step 2.
2. Add 100 μL Buffer AL. Mix thoroughly by vortexing. Incubate samples at 70°C for 10 minutes.
3. Add 200 μL 100% ethanol and mix thoroughly by vortexing.
4. Transfer the mixture into a DNeasy Mini spin column, centrifuge at 3,000 rpm for 1 minute.
5. Place the spin column into a new 2 mL collection tube, and add 500 μL Buffer AW1. Centrifuge at 6,000 rpm for 1 minute.
6. Place the spin column in a new 2 mL collection tube, and add 500 μL Buffer AW2. Centrifuge at 6,000 rpm for 1 minute.
7. Place the spin column in a new 2 mL collection tube, and centrifuge at 10,000 rpm for 1 minute.
8. Transfer the spin column to a 1.5-mL microcentrifuge tube, and elute the DNA by adding 15 μL nuclease-free water to the center of the membrane. Incubate at 37°C for 10 minutes. Centrifuge at 14,000 rpm for 2 minutes.
9. DNA concentration was measured using the NanoDrop ND-1000 Spectrophotometer.

### Data analysis

Upon completion of the Seahorse run, the final results of OCR measurement will be available in the Wave Desktop v2.6.1.5 software.

1. Normalize the OCR values for each sample based on total DNA quantity.
2. Check blank wells to ensure no false-positive OCR values are present. Deselect any erroneous values before exporting the data.
3. Review the preliminary results and identify curves with high standard deviations.
4. Navigate to “Level” under “Wells” to examine the O_2_ (mmHg) levels and inspect the curves from each well to identify and exclude possible outliers.
5. Calculate and export key bioenergetic profiles, including basal respiration, ATP-linked respiration, non-ATP-linked respiration, maximal respiration, spare respiratory capacity, and non-mitochondrial oxygen consumption.

Notes: 1. Normalizing OCR values with DNA quantity is optimal and finalized after initial normalization with tissue weight or protein quantity, both of which are unreliable or challenging due to the small size of the mouse aortic tissue. 2. Samples that fail to respond to the drugs or produce an outer layer signal, possibly due to air bubble formation or tissue movement during drug injection or mixing, can be excluded as they represent assay failures.

### Statistical analysis

Data are presented as mean ± standard deviation. Nonparametric statistical tests were conducted. Statistical differences between two groups were analyzed using unpaired Mann–Whitney analysis. For three or more groups, Kruskal–Wallis analysis was performed followed by Dunnett post-tests to compare between two specific groups. Analyses were performed using GraphPad Prism 9.0. Statistical significance was set at *P*-value <.05.

## Results

### Optimization of tissue size, drug concentrations, and incubation time, and normalization for OCR measurement in mouse aorta

**Figure 2.**
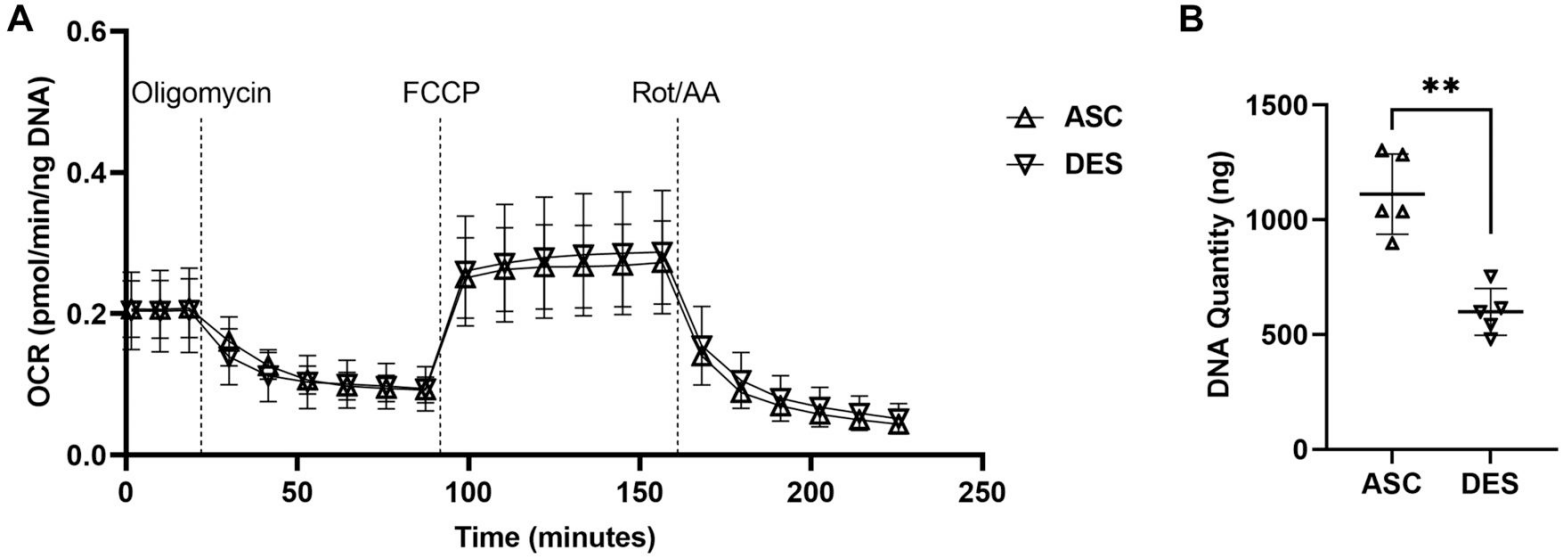
Evaluation of OCR on 2-mm ascending and descending thoracic aortic tissues from mice at the age of 28 days. A. No difference of OCR values between ascending and descending aortas (n = 5 in each group, 3 males and 2 females). B. DNA quantification analysis of the 2-mm aortic tissues after seahorse assay shows significant higher DNA quantity in the ascending aortas compared to descending aortas. FCCP, carbonyl cyanide-4 (trifluoromethoxy) phenylhydrazone; Rot, rotenone; AA, antimycin; ASC, ascending aorta; DES, descending aorta; **P < .01

Due to the small size of mouse aorta, collecting aortic tissue discs of 3 mm or 2.5 mm proved impractical in our preliminary tests, particularly from the descending aortas (**Fig. 1C**). Notably, direct comparison of unnormalized OCR values between tissue segments of different sizes from the same descending aortas shows that larger tissue size yields higher OCR values, indicating that tissue size plays a critical role in OCR measurement in mouse aortic tissues (**Supplementary Fig. 1**). Thus, 2-mm tissue discs were used consistently throughout this study. After conducting over 20 preliminary tests, the concentrations of three drugs and the wait time of each measurement cycle were optimized based on the dynamic changes in OCR values (**Supplementary Fig. 2**; **Supplementary Tables 1 and 2**). The baseline OCR value was approximately 0.2 pmol/min/ng DNA, which decreased by 50% after blocking ATP synthase with oligomycin for about 33 minutes and remained stable thereafter (**Fig. 2A**). FCCP treatment increased OCR to a stable level of 0.25-0.4 pmol/min/ng DNA within around 30 minutes (**Fig. 2A**). Rot/AA treatment induced a dramatic and continuous decline in OCR during the first three measurement cycles, ultimately reducing OCR to less than 0.1 pmol/min/ng DNA (**Fig. 2A**). Normalization of raw OCR values is critical for the data accuracy and further analysis. Using 2 mm tissue discs, we identified that DNA quantity was significant higher in ascending compared to descending thoracic aortas (**Fig. 2B****; Supplementary Fig. 3**), which reflects the different numbers of cells in the aortic wall, particularly medial smooth muscle cells in wild-type mice. This finding is supported by the greater number of lamellar layers and associated cell numbers in the ascending aortas in various mammals [21–23]. Consequently, we normalized OCR values based on DNA quantity for this aortic tissue analysis.

### No sex or regional differences of OCR in mouse aorta

After validating the responses of mouse aortas to various drugs and optimizing the timing of measurements, we measured OCR in aortas from both male and female mice. The results revealed no significant differences in OCR between the ascending and descending aortas in either sex (**Supplementary Fig. 4**). Importantly, DNA quantity was higher in ascending aortas compared to descending aortas in both male and female mice, with no differences between the sexes (**Supplementary Fig. 4**). Consequently, tissue samples from both sexes were included in subsequent assays. The OCR values of ascending aortic tissues did not differ significantly from those of descending aortas (**Fig. 2A****; Supplementary Fig. 3**). To validate these findings, we assessed aortic tissues at two different time points, P32 and 10 weeks of age (**Supplementary Figs. 5 and 6**). The results were consistent with those observed in P28 aortic tissues, confirming the same pattern.

## Discussion

Since its introduction in 2006, Seahorse extracellular flux technology has become widely used in cellular metabolism research, offering the advantage of minimizing stress from single-cell preparation and enabling the assessment of the metabolic state of cells [24]. Recently, this technology has been extensively used to evaluate near-natural metabolic profiles in tissues from various systems [10–17]. With the growing recognition of mitochondrial dysfunction in the progression of aortic diseases [4–6], there is a pressing need for direct analysis of mitochondrial function in aortic tissue. In this study, we adapted this technology to analyze intact aortic tissues, building on previous reports [11]. We developed a reliable and reproducible method for measuring OCR by making significant modifications on tissue size, drug concentrations, and drug incubation time.

Baseline conditions, including drug concentrations and timing of each measurement cycle, are adapted based on our *in vitro* assay, with oligomycin, FCCP, and Rot/AA concentrations set at 1 µM, 1.5 µM, and 1 µM, respectively [25]. Initially, the intact aortic tissues show only mild and slow changes in OCR, indicating inadequate responsiveness. To address this, we made adjustments to ensure full drug penetration into the aortic tissue, enabling the capture of the dynamic flux of oxygen in the surrounding medium. The effectiveness of drug induction is evidenced by steady changes in OCR during the first three measurements, followed by a stabilized OCR in the subsequent three measurements after each drug injection. Notably, increasing the FCCP concentration to 6 µM does not alter the maximum OCR compared to 4.5 µM. Additionally, extending the wait or drug incubation time beyond 10 minutes in each measurement cycle results in a final stable OCR value without any increase or decline in the curve. These dynamic changes are critical for demonstrating full drug penetration and cellular responses to treatment. Therefore, we included six measurement cycles in the final protocol.

The levels of OCR values are correlated with the number of cells in the analyzed tissues, making normalization crucial to minimize noise and errors introduced during sample preparation. Initially, we attempted to measure the weight [16] of mouse aortic tissues, both wet and dry, but this did not yield reliable results. Direct weighing often recorded 0 mg, while indirect methods sometimes produced negative values, underscoring the challenges of accurately quantifying tissue mass in such small samples. Subsequently, total protein [12, 15] was extracted from ascending and descending aortic segments, but tissue or solution loss during fragmentation and sonication posed a major challenge for absolute quantification. As no significant differences in protein levels were detected between 2 mm tissue discs, OCR curves remained unchanged after normalization (**Supplementary Fig. 7**). Additionally, extracellular matrix accumulation of proteoglycans is a major histopathological change during aortic disease progression, normalizing OCR by total protein content could lead to significant errors in interpreting metabolic changes within live cells. Therefore, we employed a standard DNA quantification protocol. The DNA quantity [14] in different regions shows a pattern consistent with previous histological studies [21–23], making it a reliable normalization method. To further enhance the reliability of this protocol, we standardized the use of 2-mm aortic tissue punches. This approach not only minimizes variations in all captured parameters but also serves as a reliable data quality control measure.

In addition to the tests mentioned above, tissue harvest time, screen orientation, and tissue orientation were evaluated during the pretests. Harvesting tissue for longer than 2 h with the current medium leads to a significant decrease in baseline OCR, likely due to reduced cell viability. Notably, the screen orientation is reversed to minimize potential physical compression of the aortic tissue, as the depth of the tissue container at the bottom of the well in the islet plate is only 200 µm. This modification also allows testing in disease-affected aortas, as significant thickening and remodeling can occur under disease conditions. Finally, tissue orientation, whether the intima faces up or down, does not impact the OCR values.

A limitation of this protocol, as with all tissue analysis methods, is that it cannot be used to assess the OCR of specific cell types within the tissue. However, it provides valuable data on the predominant cell types at baseline, such as smooth muscle cells in the aortic media. The progression of thoracic aortic disease involves increased tissue thickness as well as the infiltration and proliferation of various cell types, including macrophages and fibroblasts, within the aortic wall. Further specialized analyses are required to characterize the specific OCR changes in these cells.

In summary, we report an optimized method using the Agilent Seahorse XFe24 analyzer to measure mitochondrial respiration in mouse aorta, which enables accurate evaluation of mitochondrial changes within the tissue. This method provides a foundational framework for further investigations into mitochondrial function under disease conditions.

## Supplementary Material

bpaf031_Supplementary_Data

## Data Availability

The data underlying this article will be shared on reasonable request to the corresponding authors.
