# Supplementary material for: Measurement of oxygen consumption rate in mouse aortic tissue
Source: Biol Methods Protoc. 2025 Apr 24;10(1):bpaf031. doi: 10.1093/biomethods/bpaf031 (PMC12054972; doi:10.1093/biomethods/bpaf031)
Supplement: bpaf031_Supplementary_Data [file bpaf031_supplementary_data.zip › Supplementary Material.pptx]

## Slide 1
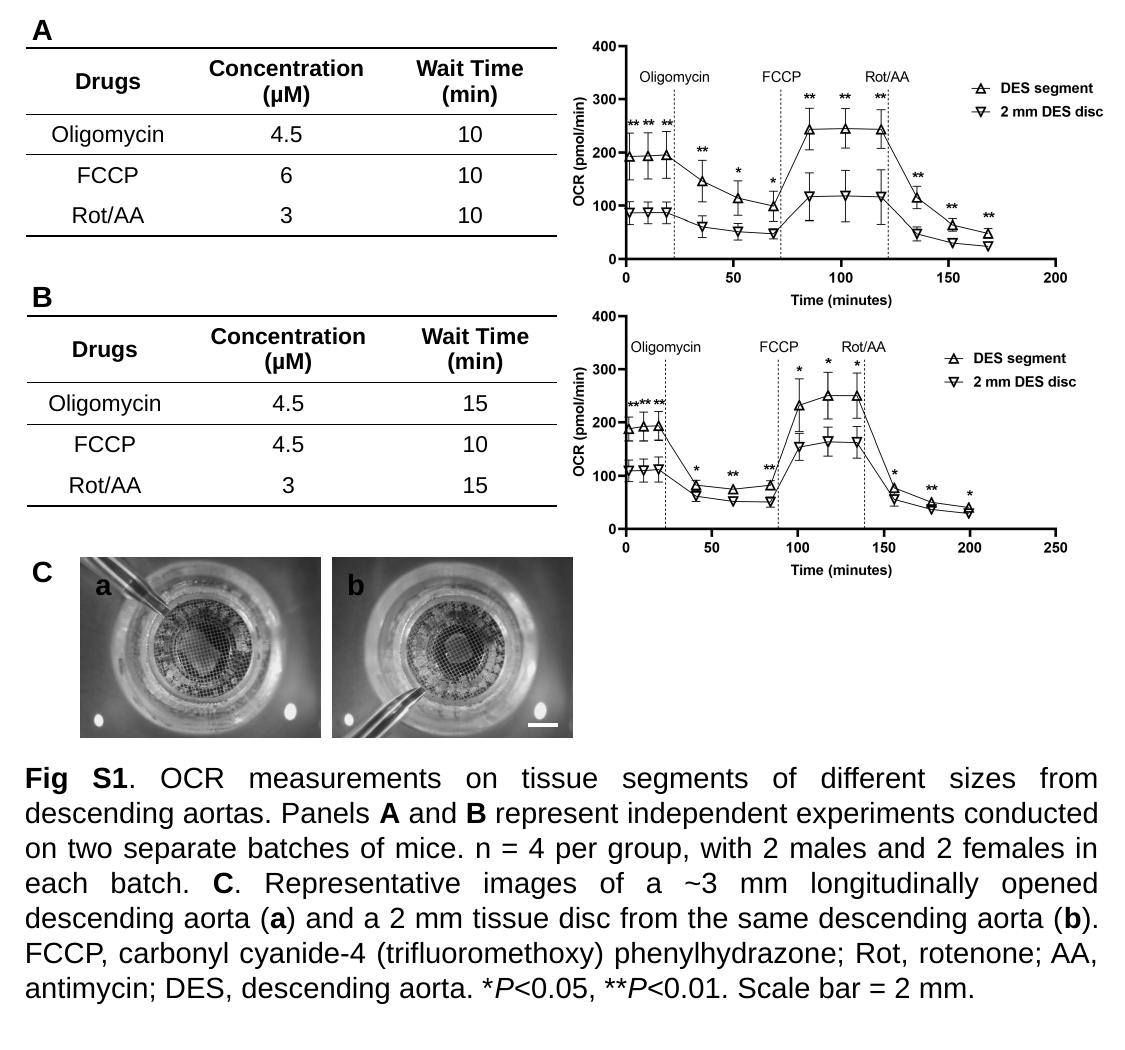

A
| Drugs | Concentration (µM) | Wait Time (min) |
| --- | --- | --- |
| Oligomycin | 4.5 | 10 |
| FCCP | 6 | 10 |
| Rot/AA | 3 | 10 |
B
| Drugs | Concentration (µM) | Wait Time (min) |
| --- | --- | --- |
| Oligomycin | 4.5 | 15 |
| FCCP | 4.5 | 10 |
| Rot/AA | 3 | 15 |
C
a
b
Fig S1. OCR measurements on tissue segments of different sizes from descending aortas. Panels A and B represent independent experiments conducted on two separate batches of mice. n = 4 per group, with 2 males and 2 females in each batch. C. Representative images of a ~3 mm longitudinally opened descending aorta (a) and a 2 mm tissue disc from the same descending aorta (b). FCCP, carbonyl cyanide-4 (trifluoromethoxy) phenylhydrazone; Rot, rotenone; AA, antimycin; DES, descending aorta. *P<0.05, **P<0.01. Scale bar = 2 mm.

## Slide 2
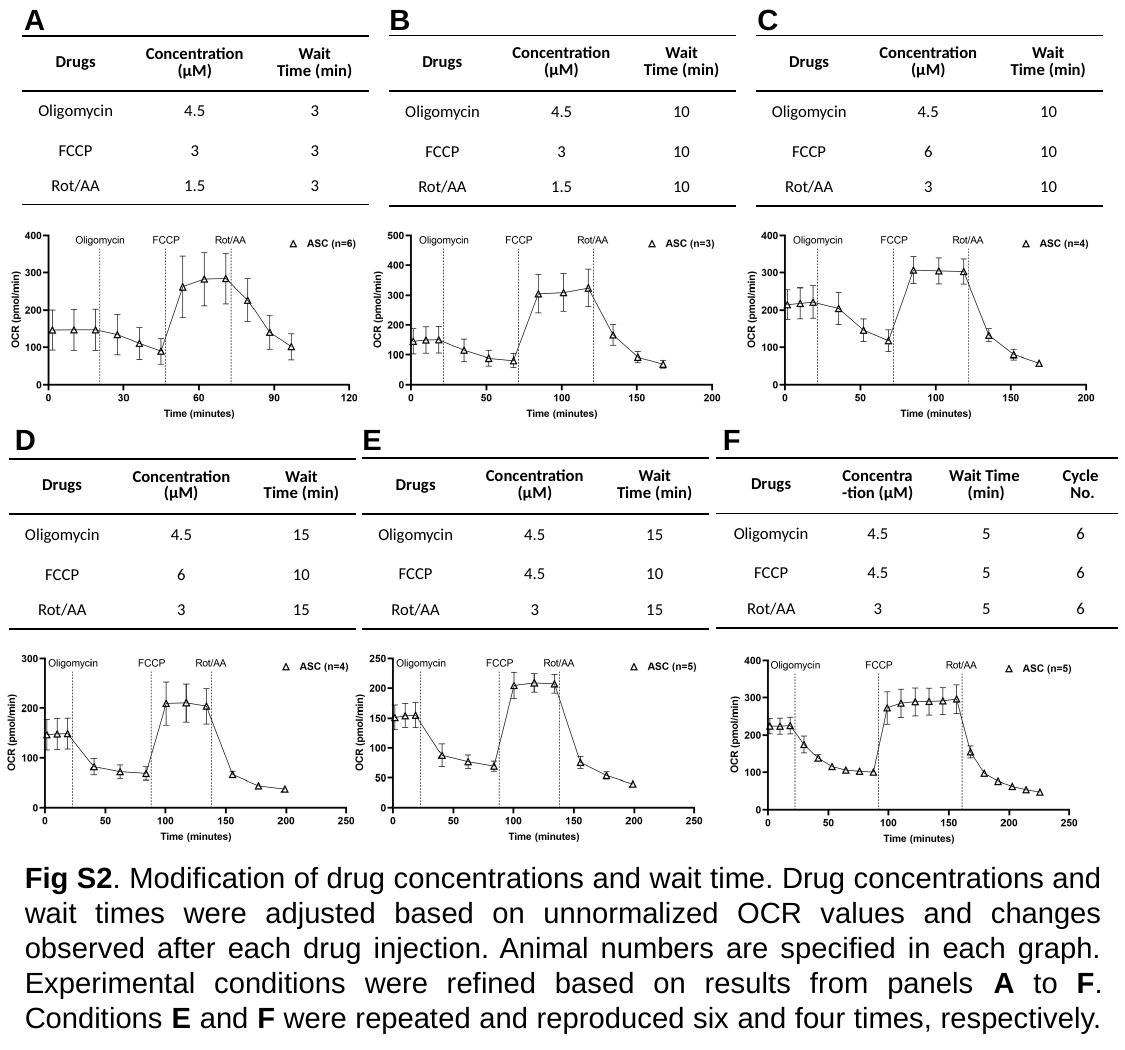

A
B
C
| Drugs | Concentration (µM) | Wait Time (min) |
| --- | --- | --- |
| Oligomycin | 4.5 | 10 |
| FCCP | 3 | 10 |
| Rot/AA | 1.5 | 10 |
| Drugs | Concentration (µM) | Wait Time (min) |
| --- | --- | --- |
| Oligomycin | 4.5 | 10 |
| FCCP | 6 | 10 |
| Rot/AA | 3 | 10 |
| Drugs | Concentration (µM) | Wait Time (min) |
| --- | --- | --- |
| Oligomycin | 4.5 | 3 |
| FCCP | 3 | 3 |
| Rot/AA | 1.5 | 3 |
D
E
F
| Drugs | Concentra-tion (µM) | Wait Time (min) | Cycle No. |
| --- | --- | --- | --- |
| Oligomycin | 4.5 | 5 | 6 |
| FCCP | 4.5 | 5 | 6 |
| Rot/AA | 3 | 5 | 6 |
| Drugs | Concentration (µM) | Wait Time (min) |
| --- | --- | --- |
| Oligomycin | 4.5 | 15 |
| FCCP | 4.5 | 10 |
| Rot/AA | 3 | 15 |
| Drugs | Concentration (µM) | Wait Time (min) |
| --- | --- | --- |
| Oligomycin | 4.5 | 15 |
| FCCP | 6 | 10 |
| Rot/AA | 3 | 15 |
Fig S2. Modification of drug concentrations and wait time. Drug concentrations and wait times were adjusted based on unnormalized OCR values and changes observed after each drug injection. Animal numbers are specified in each graph. Experimental conditions were refined based on results from panels A to F. Conditions E and F were repeated and reproduced six and four times, respectively.

## Slide 3
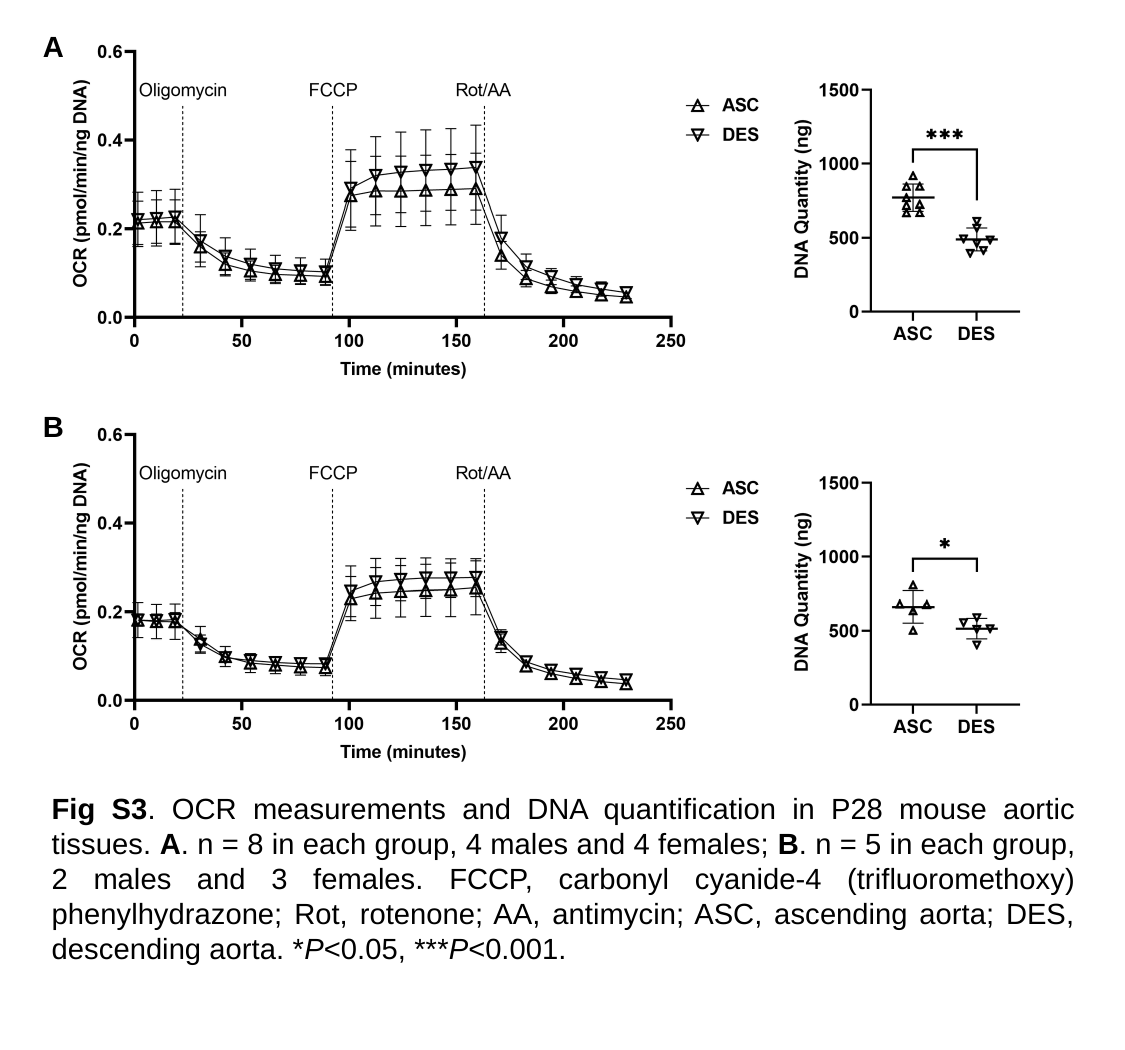

A
B
Fig S3. OCR measurements and DNA quantification in P28 mouse aortic tissues. A. n = 8 in each group, 4 males and 4 females; B. n = 5 in each group, 2 males and 3 females. FCCP, carbonyl cyanide-4 (trifluoromethoxy) phenylhydrazone; Rot, rotenone; AA, antimycin; ASC, ascending aorta; DES, descending aorta. *P<0.05, ***P<0.001.

## Slide 4
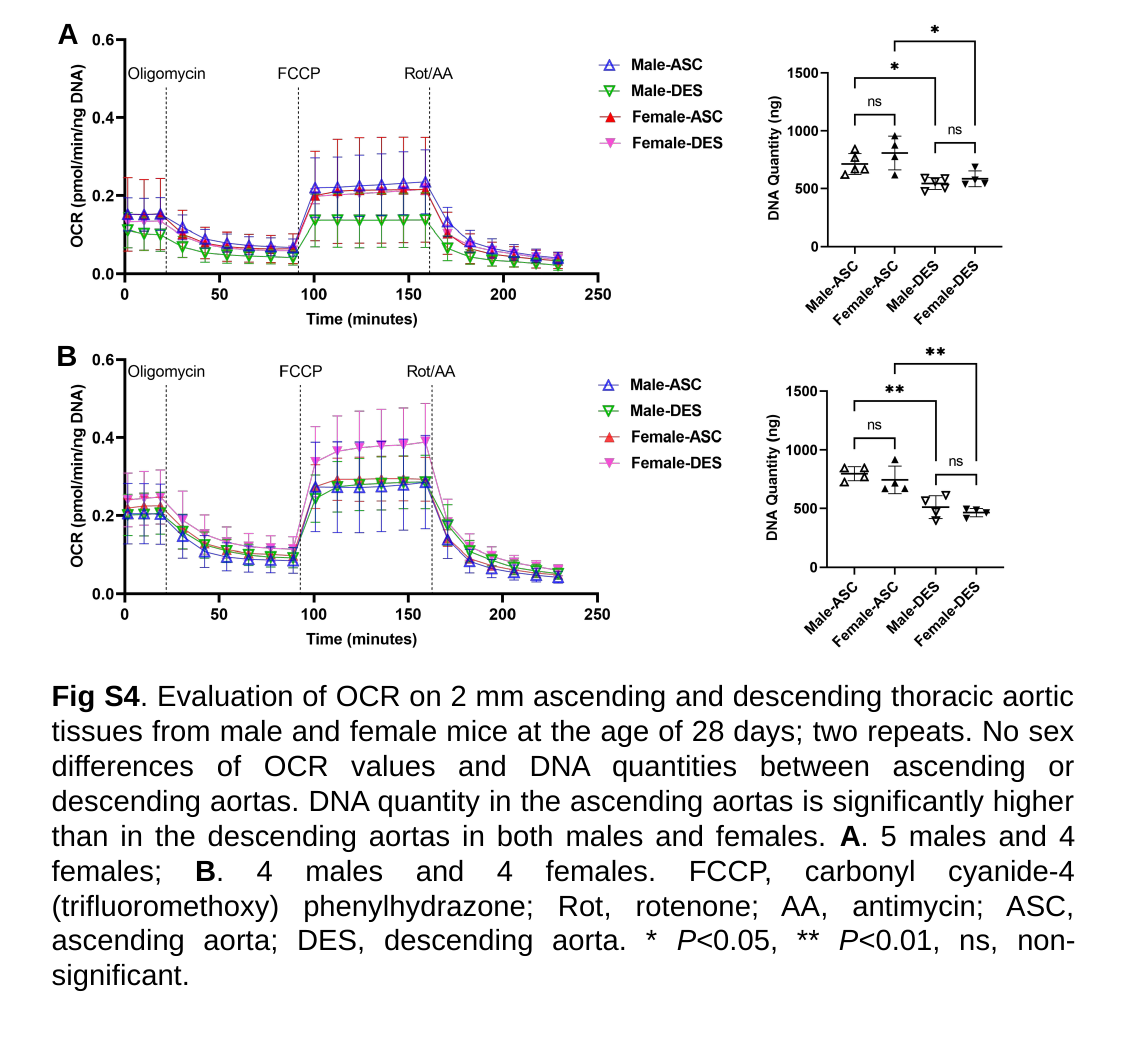

A
B
Fig S4. Evaluation of OCR on 2 mm ascending and descending thoracic aortic tissues from male and female mice at the age of 28 days; two repeats. No sex differences of OCR values and DNA quantities between ascending or descending aortas. DNA quantity in the ascending aortas is significantly higher than in the descending aortas in both males and females. A. 5 males and 4 females; B. 4 males and 4 females. FCCP, carbonyl cyanide-4 (trifluoromethoxy) phenylhydrazone; Rot, rotenone; AA, antimycin; ASC, ascending aorta; DES, descending aorta. * P<0.05, ** P<0.01, ns, non-significant.

## Slide 5
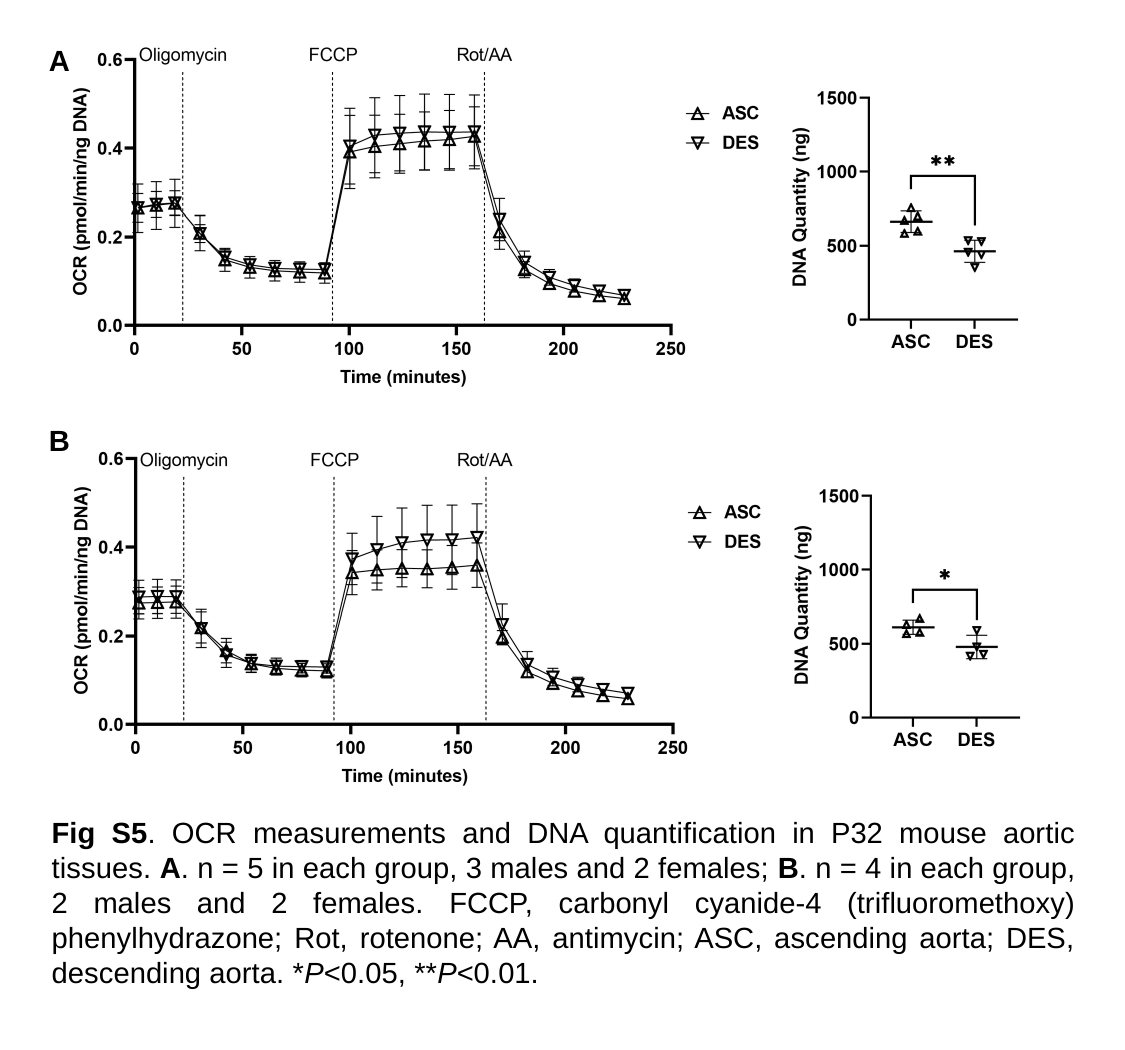

A
B
Fig S5. OCR measurements and DNA quantification in P32 mouse aortic tissues. A. n = 5 in each group, 3 males and 2 females; B. n = 4 in each group, 2 males and 2 females. FCCP, carbonyl cyanide-4 (trifluoromethoxy) phenylhydrazone; Rot, rotenone; AA, antimycin; ASC, ascending aorta; DES, descending aorta. *P<0.05, **P<0.01.

## Slide 6
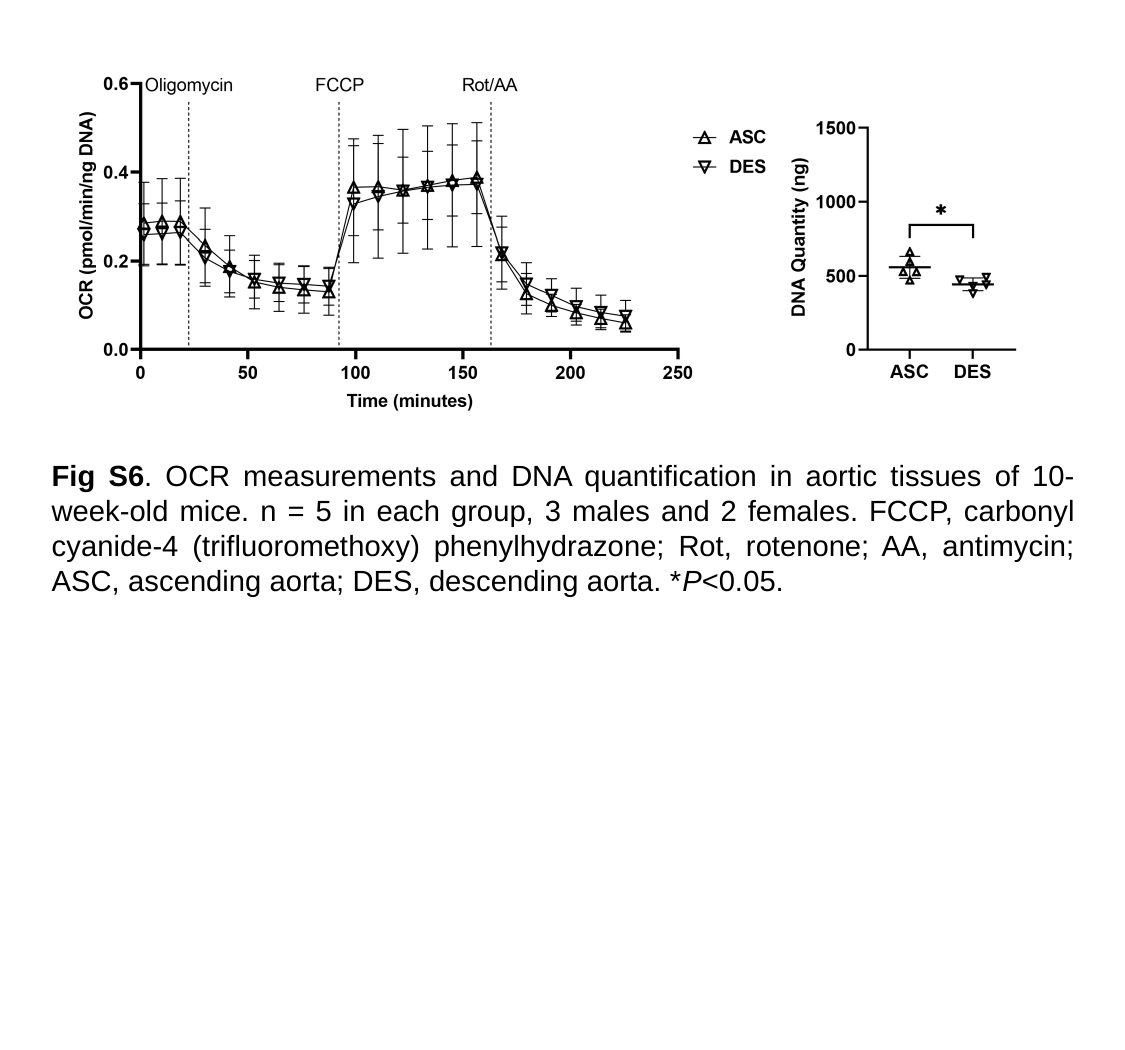

Fig S6. OCR measurements and DNA quantification in aortic tissues of 10-week-old mice. n = 5 in each group, 3 males and 2 females. FCCP, carbonyl cyanide-4 (trifluoromethoxy) phenylhydrazone; Rot, rotenone; AA, antimycin; ASC, ascending aorta; DES, descending aorta. *P<0.05.

## Slide 7
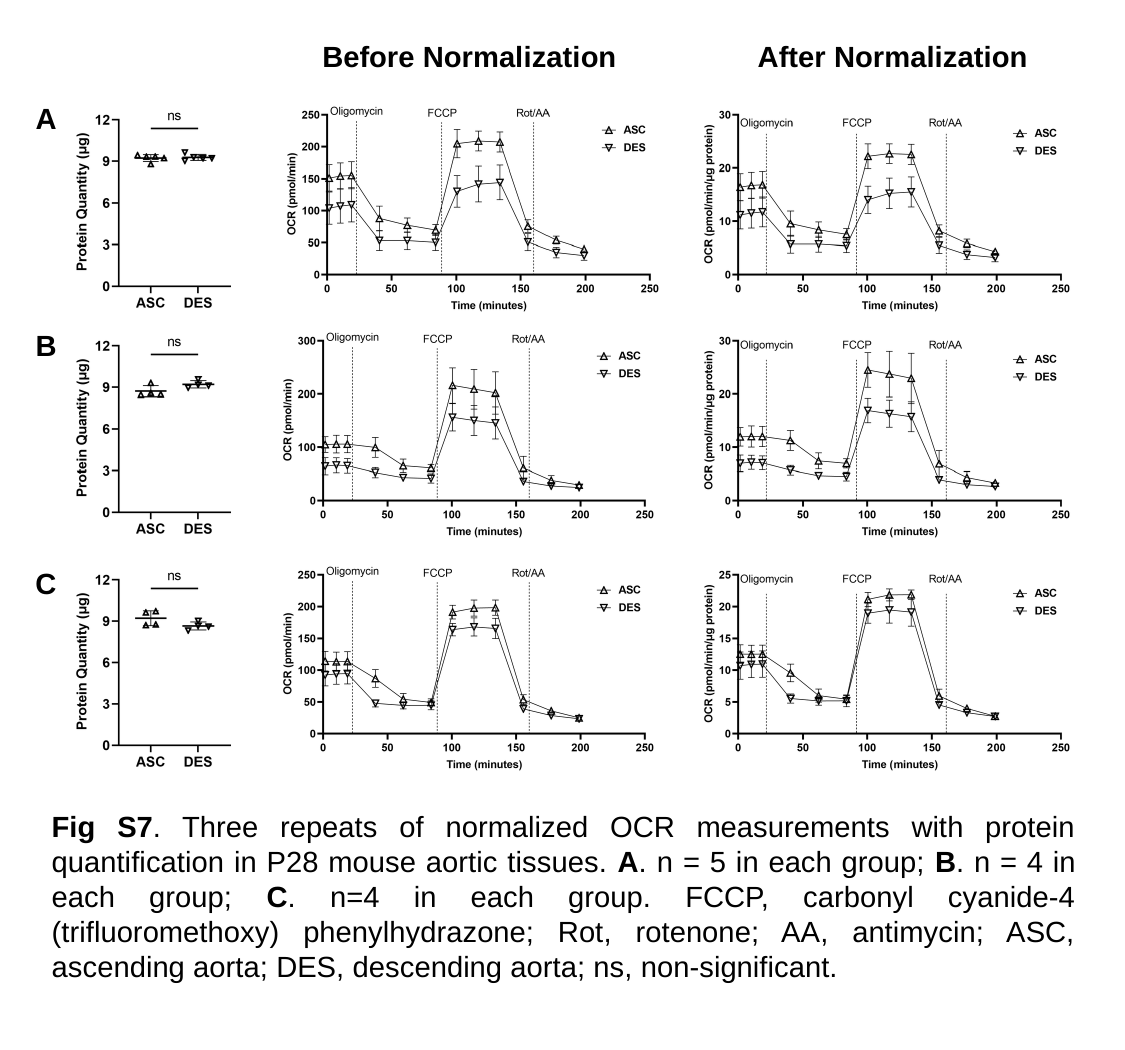

Before Normalization
After Normalization
A
B
C
Fig S7. Three repeats of normalized OCR measurements with protein quantification in P28 mouse aortic tissues. A. n = 5 in each group; B. n = 4 in each group; C. n=4 in each group. FCCP, carbonyl cyanide-4 (trifluoromethoxy) phenylhydrazone; Rot, rotenone; AA, antimycin; ASC, ascending aorta; DES, descending aorta; ns, non-significant.

## Slide 8
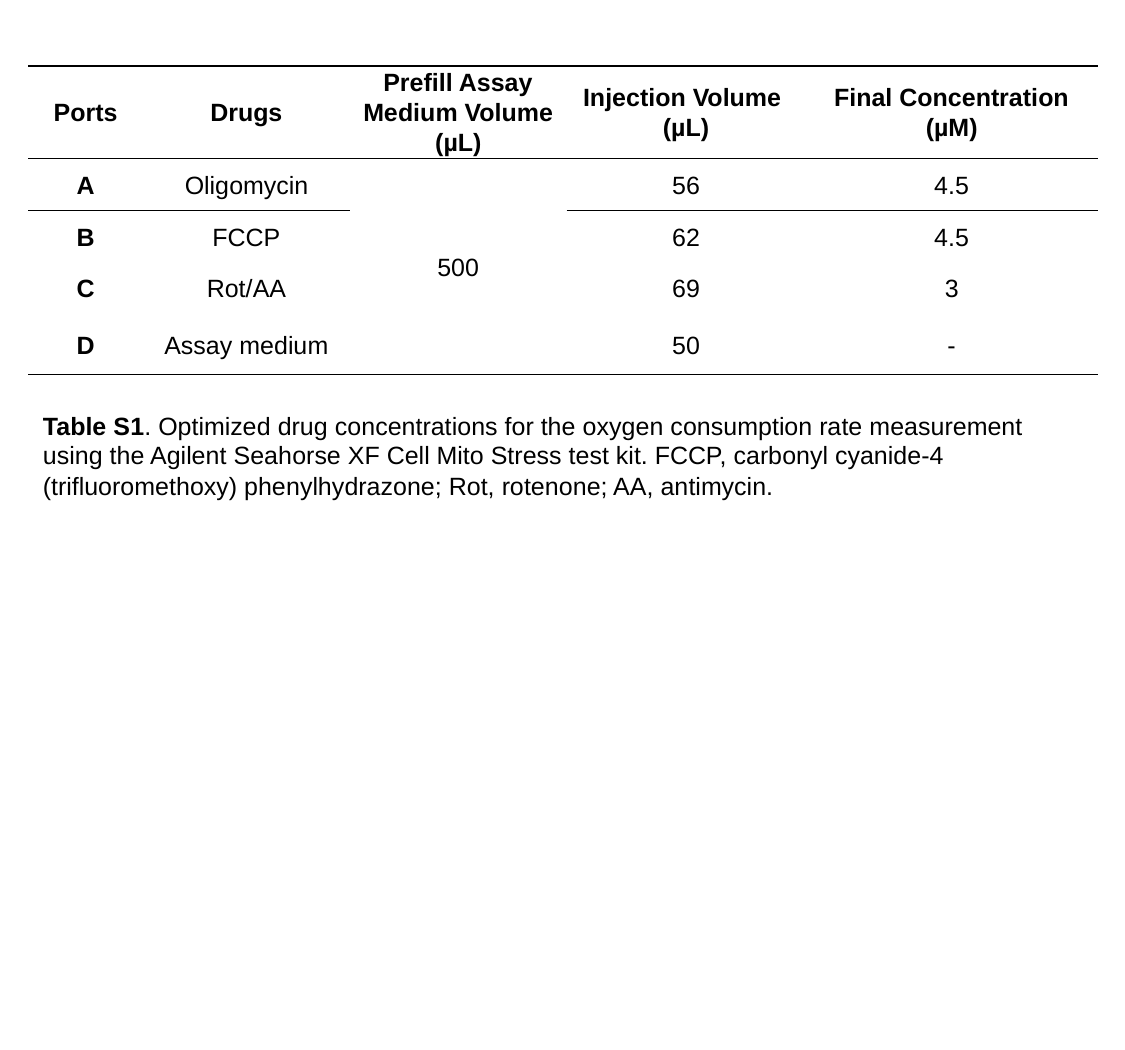

| Ports | Drugs | Prefill Assay Medium Volume (µL) | Injection Volume (µL) | Final Concentration (µM) |
| --- | --- | --- | --- | --- |
| A | Oligomycin | 500 | 56 | 4.5 |
| B | FCCP | | 62 | 4.5 |
| C | Rot/AA | | 69 | 3 |
| D | Assay medium | | 50 | - |
Table S1. Optimized drug concentrations for the oxygen consumption rate measurement using the Agilent Seahorse XF Cell Mito Stress test kit. FCCP, carbonyl cyanide-4 (trifluoromethoxy) phenylhydrazone; Rot, rotenone; AA, antimycin.

## Slide 9
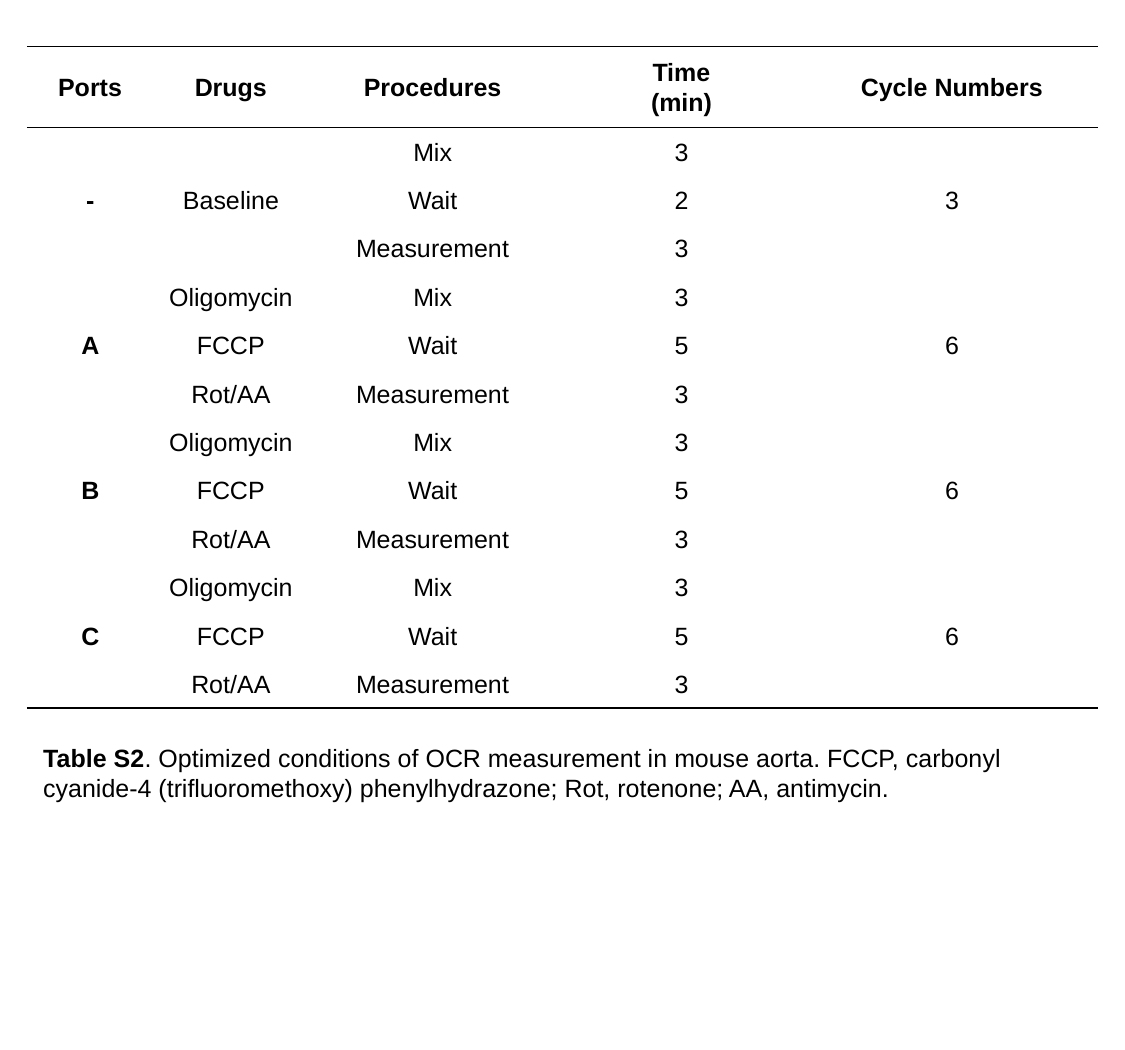

| Ports | Drugs | Procedures | Time (min) | Cycle Numbers |
| --- | --- | --- | --- | --- |
| - | Baseline | Mix | 3 | 3 |
| | | Wait | 2 | |
| | | Measurement | 3 | |
| A | Oligomycin | Mix | 3 | 6 |
| | FCCP | Wait | 5 | |
| | Rot/AA | Measurement | 3 | |
| B | Oligomycin | Mix | 3 | 6 |
| | FCCP | Wait | 5 | |
| | Rot/AA | Measurement | 3 | |
| C | Oligomycin | Mix | 3 | 6 |
| | FCCP | Wait | 5 | |
| | Rot/AA | Measurement | 3 | |
Table S2. Optimized conditions of OCR measurement in mouse aorta. FCCP, carbonyl cyanide-4 (trifluoromethoxy) phenylhydrazone; Rot, rotenone; AA, antimycin.
